# Development of a new staining protocol for the Kleihauer–Betke test to facilitate the reading of difficult cases

**DOI:** 10.1186/s12884-024-06258-9

**Published:** 2024-01-29

**Authors:** Adrian Serban, Yannick Tholance, Carmen Aanei, Lydia Campos, Cristina Iobagiu

**Affiliations:** 1 Laboratory of Biochemistry, University Hospital of Saint-Etienne, Saint-Priest-en-Jarez, 42275 France; 2 Institute NeuroMyoGène, INSERM U1217/CNRS UMR 5310, Université Claude Bernard Lyon 1, Lyon, France; 3 Laboratory of Hematology, University Hospital of Saint-Etienne, Saint-Priest-en-Jarez, 42275 France; 4 INSERM U1059-SAINBIOSE, Université de Lyon, Saint-Etienne, France; 5 Immunohematology Laboratory, Etablissement Français de Sang, Saint-Etienne, France

**Keywords:** Kleihauer, Betke test, New staining protocol, False positive, Hemoglobin electrophoresis, Comparative study

## Abstract

**Background:**

The Kleihauer–Betke (KB) test allows the detection of fetal red blood cells (containing fetal hemoglobin, HbF) in the maternal blood to identify and quantify potential fetal-maternal hemorrhages. In certain cases, detecting fetal red blood cells with conventional staining is difficult. False-positive results or overestimation of the quantity of fetal red blood cells may occur in cases of maternal hemoglobinopathy. In this study, we developed a new staining protocol to facilitate the reading of difficult smears and improve the precision of the quantification of fetal red blood cells; we also analyzed the performance of this new method. This study assessed blood samples with and without hemoglobin abnormalities, which present difficulties when interpreting the KB test.

**Methods:**

The new staining formula is based on an improved elution technique and the use of a different stain instead of hematoxylin. To test this staining method, 16 samples from patients with abnormal hemoglobin electrophoresis and 14 samples from patients with normal hemoglobin electrophoresis were analyzed using the KB test with the classical staining method and the new staining method. In addition, a second series was prepared using the same samples spiked with fetal red blood cells from newborn blood, to compare the accuracy of the two methods in identifying fetal red blood cells.

**Results:**

In the 60 slides analyzed with both staining methods, we found that the new technique improved the accuracy from 78 to 85%; lowered the coefficient of variation between the operators, which decreased from 20.7% to 12.7%; increased the specificity in our population from 56 to 70%; and decreased the number of false-positive cases by 30%.

**Conclusions:**

We successfully developed a new staining technique that facilitates the reading of difficult slides and improves the specificity of the detection of fetal red blood cells. This technique is recommended as a secondary method to use before sending the sample for additional exploration.

**Supplementary Information:**

The online version contains supplementary material available at 10.1186/s12884-024-06258-9.

## Introduction

The Kleihauer–Betke test (KB) was introduced in 1957 in response to the clinical necessity of fetal-maternal bleeding detection [1]. The primary concern is the Rhesus (Rh) incompatibility between a mother who is Rh-negative and a fetus that is Rh-positive because the mixing of the fetal and maternal blood produces an alloimmunization of the mother.These immunoglobulins (IgGs) can pass through the placental barrier [2] and lead to a hemolytic disease in the newborn [3]. All pregnant women who are Rh-negative typically receive Rhesus immune globulin as prophylaxis against anti-D-alloimmunization according to guidelines [4]. In addition, in cases of feto–maternal bleeding, the previously administered Rhesus Imune globuline can become insufficient; therefore, the quantification of hemorrhage severity is useful for adjusting the dose of Rhesus immune globulin [3]. The second concern relates to trauma, the KB can accurately predict the risk of preterm labor after maternal trauma, and the study [5] assure that the cases with a negative KB, post-trauma monitoring can be safely limited.

The test’s main principle relies on the acid resistance of fetal hemoglobin (HbF) [1]. Because the original technique was laborious and time-consuming, the Shepard method was developed, offering a faster stain production [6]. Moreover, this method was more sensitive than the original method, with a sensibility of 1 fetal erythrocyte per 20,000 adult erythrocytes, which can be safely scored as nonsignificant bleeding. As in response to variation of blood cell properties and characteristics, the Mollison formula is currently used as a good correlation between the KB result and the mL of hemorrhage [7, 8].

Sometimes, the slides contained ambiguous RBCs that could not be easily classified as fetal or adult cells, making the counting very difficult. This phenomenon still occurred when the slide preparation procedure was repeated, and it has been described previously in the literature [9, 10]. The irregular staining of certain RBCs may be due to different types of HbF, as described in [11], or cases of hemoglobin diseases, such as thalassemia or sickle cell disease, as described in [12].

There is now a reference technique for detecting fetal RBCs in cases of suspected fetal-maternal hemorrhage: flow cytometry. This method uses the specific antibody anti-hemoglobin F coupled with fluorescein to detect fetal RBCs [13, 14]. Unfortunately, this technique is expensive and time-consuming, and its use is limited to doubtful cases.

The purpose of our study was to adapt the staining protocol to facilitate the interpretation of slide reading in difficult cases. We also analyzed the global performance of the new staining method and compared it with the Shepard technique on a population with a high percentage of hemoglobin anomalies. We developed a staining protocol that facilitates the operator’s reading and counting of the slide, reduces the number of false-positive results, and is suitable for use with automatic techniques of microscopy.

## Material and methods

### Study design and selection criteria of patients

This study evaluated the performance of a new KB staining test on a nonpregnant population with a high rate of hemoglobin abnormalities and compared the results with those obtained with the Shepard technique currently used in our laboratory. We ensured that none of the patients included in the study were pregnant by verifying in the medical files the possibility of a pregnancy or the recent history of a negative total human chorionic gonadotropin (HCG) test.

We selected patients for whom the results of hemoglobin electrophoresis and blood grouping were available from December/29/2020 to February/04/2021.The samples were provided by the laboratory of biochemistry in the CHU Saint Etienne, and represents the blood that remains after the prescribed analysis has been carried out. Sixteen O + patients, one O − patient, nine A + patients, and four B + patients aged between 18 and 79 years old met these criteria. The blood samples were used respecting the conservation time of a maximum of 1 month at + 4 °C in a biochemistry laboratory.

As a positive control to evaluate the fetal RBC counting accuracy, we spiked in the patient samples newborn blood of phenotype O obtained from the EFS used currently as a reagent.

The newborn blood was diluted with the adult blood at a volume ratio of 1/100 to achieve approximately 140 fetal RBCs to 10,000 adult RBCs. The smears were carried out on the same day that the newborn mixture was prepared, according to the procedure currently used in the immunohematology laboratory. Each patient had four smears: two native smears, expected negative (without fetal RBCs) and two smears containing the newborn/patient blood mixture, expected positive. One negative and one positive smear were stained using the Shepard method, and two others smears were stained using the new method, described above. Therefore, the drying time was the same for each couple of slides.

To obtain the number of fetal RBCs in each mixture preparation, we used data from complete blood count. We divided the RBC count per mL of newborn blood used to prepare the mixture by the RBC count per mL of the patient, ratio (newborn RBC/adult RBC) multiplied by 100, resulting in a reference value for accuracy evaluation.

### Staining technique

The blood samples for KB test are diluted 1/3 in physiologic serum. The smears are performed and dried for a minimum of 30 min. For each smear, exactly 5 µL of diluted blood was used. We standardized this quantity because in previous observations (data not shown), the elution was reversed proportionally with the quantity of blood on the slide.

**Fig. 1:**
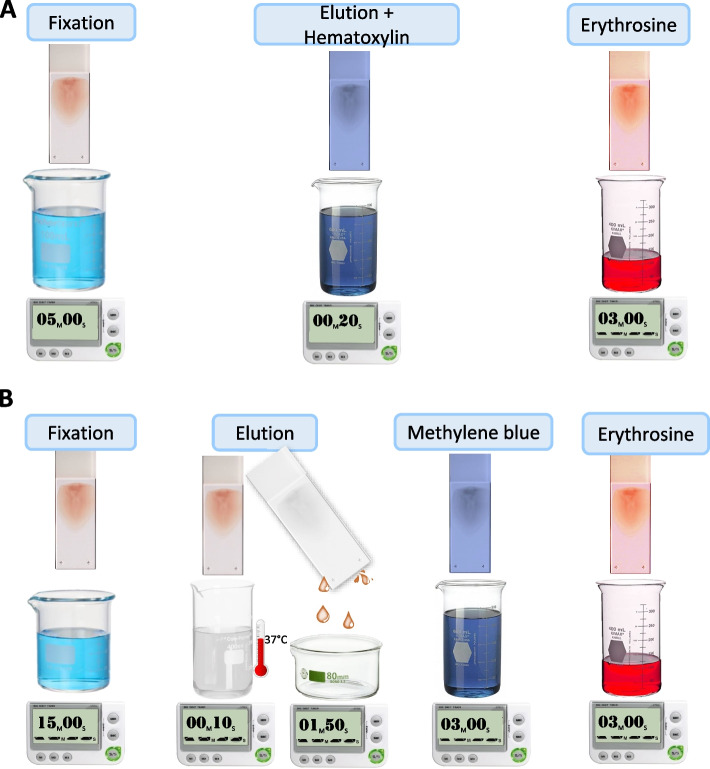
Representation of the two staining protocols. **A** Description of the Shepard staining **B** description of the proposed staining protocol

In the Shepard technique (Fig. 1A), the slides arranged in a slide bank are immersed for 5 min in a fixation solution containing 80% ethanol. After a drying step, they are immersed in a solution of iron, chlorohydric acid, and hematoxylin for 20 s. In this step, the slides are eluted by the acidic environment, and the acidic cellular structures are stained blue by hematoxylin. After washing with tap water, the bank of slides was immersed in the 1% erythrosine solution for red staining of the alkaline compounds present in the cells, such as noneluted hemoglobin and the cell membrane.

For the new method (Fig. 1B), we considered a fixation of 15 min to be acceptable, using the same 80% alcoholic solution used in the Shepard method. This fixation is shorter than 60 min used in the original KB technique, and did not remove the cells from the slide by the acid, which could disrupt the ratio adult/fetal RBCs. After, we used two steps for elution and hematoxylin staining. The elution is the most important step in this technique, and a substantial number of experiments and outcomes were required to determine the best formula. To acquire simple reactant solutions that were easy to find and reasonably priced, we chose to make a solution of acetic acid 1/1000, using 0.9% NaCl and 0.2% bovine serum albumin solution. This solution’s pH was measured with an Accumet AB 150 (produced by Fisherbrand™) and was determined to be 3.09, adequate for an effective elution. We also chose to retain the original temperature recommendation for the eluent (37 °C).

In fact, we tested a theory relying on the osmolarity gradient. The fixation was performed with an alcoholic solution, so the cells were fixed at their original size and dehydrated after the drying step. The hyperosmotic acid solution, measured at 530 mOsm/kg, leads to protein denaturation, normally contributing to cell fixation. The acid penetrates the cell, solubilizes and denatures the hemoglobin, and facilitates the hemoglobin wash. In the wash step, which is performed with deionised water measured at 0 mOsm/kg, the cells that contain hemoglobin become larger because the water is retained in the cell by osmotic forces, and the eluted cells, which do not have as much osmotic potential inside, become smaller. This process facilitates the elution and the creation of a size difference between the eluted and non-eluted cells.

In the final formula, bovine serum albumin acted as a membrane stabilizer, and the acetic acid, even if it forms dimers, easily crossed the membrane barrier to react with hemoglobin. Ten seconds was considered adequate for slide immersion, but we allowed the reaction to continue for another 90–120 s before washing, with the slide slightly angled to facilitate fluid movement. In the 10-s immersion step, the acid enters the cells; in the reaction step, the adult hemoglobin is solubilized, and a red–brown liquid running down the slide can be seen, confirming that the elution worked. The slide is then washed under tap water for at least 10 s. The advantage of this method is a considerable decrease in the volume of elution solution used; this is because most of the eluted hemoglobin is removed during the wash, so it does not contaminate the existing solution. With this modification, we expect a more efficient slide elution with less hemoglobin residue along with an increased stability time of the reactant and more uniform slide results. It must be noted that the elution is directly dependent on the smear thickness (to obtain reproducible results, all the smears must have the same cellular load).

The next step is the blue staining, for which the classical methods use hematoxylin. One of the problems is the poor staining of eluted cell membranes and white cells, especially lymphocytes, which have the same dimensions as fetal erythrocytes, causing these cells to easily be misclassified [3]. We conducted experiments with May Grunwald Giemsa (MGG) stain applied to an eluted smear using the staining protocol used in the hematology laboratory. The white blood cells were more visible, but the fetal cells became grey and were barely visible on the slide. Therefore, the two methods were combined by mixing the methylene blue and still using the erythrosine at the end. We expect that methylene blue will reduce errors due to inexperienced users confusing fetal RBCs with small nucleated cells. Our preferred incubation time was 3 min, but the color contrast and intensity can be adjusted according to the operator’s preferences. The washing step after the staining is very important; it must be performed under tap water for at least 10 s.

The last step of the staining is the same as that in the Shepard method: 3 min of erythrosine followed by a wash. The slide reading is performed using a microscope with a blue filter and the condenser set to 2/10.

### Smear reading data collection

Fetal RBC counting was performed under an optical microscope with 40 × magnification and an ocular lens grid with 100 squares. We counted the number of adult RBCs on the 10 squares (comprising the diagonal) and the fetal cells in 100 squares. We repeated this procedure fifty times and subsequently estimated the number of adult RBCs between 10,000 and 20,000 [7]. To calculate the final result, we multiplied the total number of fetal RBCs in 5000 squares by 100 and divided it by the number of all adult RBCs in 500 squares; the result was the number of fetal cells observed per 10,000 adult RBCs. The counting of each smear was recorded on paper and the final result was integrated into an Excel sheet.

Two operators read all 120 slides. The four slides for each patient were grouped together, and they were read in the following order: None of the slides was labeled with the pathology of the patient, and the order of the patient’s reading was not important. Each operator was blinded to the results obtained by the other. Before each reading, all operators were assessed for eye accommodation using a positive case.

All the slides were classified as positive or negative using the threshold of 5 fetal RBCs/10,000 adult RBCs. The average of the two counts and the variation coefficient between the two slide readings was calculated. Using the expected values of fetal RBC in the blood mixture, we were able to evaluate the accuracy of each fetal RBC count with the two staining methods. We calculate the median of these accuracy values and of the standard deviation, for the two techniques, for comparison purpose and we evaluate the sensitivity and the specificity of each staining method. Quantitative data of fetal cells reported to 10,000 adult RBC for the 30 patients are presented as medians (quartile 1 [Q1]–quartile 3 [Q3]) (as the data did not follow a normal distribution). In addition, considering the hemoglobin electrophoresis results for each patient, the correlation between the value of the ambiguous RBCs and the percentage of the hemoglobin other than HbA was assessed.

### Statistics

The statistical data were analyzed using R software produced by the CRAN project, version i386 4.1.2. We used nonparametric tests for comparison, such as the Wilcoxon test, and the correlation was assessed by Spearman correlation. The average, median, quartiles and standard deviation were calculated in the Microsoft Office Excel (version 2016).

## Results

### Description of population

Our study included 30 patients: 16 patients had abnormalities in hemoglobin electrophoresis with variable pathologies and 14 patients had normal hemoglobin electrophoresis (including five patients with iron deficiency, confirmed by a significant decrease in ferritin). All the demographic and the hematological characteristics of the normal patients and the patients with hemoglobin abnormalities are described and compared in Table 1. The two groups were matched in term of age and sex (*p* = 0.966 and *p* = 0.712, respectively). In addition, no significant differences in the parameters of the complete blood count were observed. The five patients with iron deficiency had a mean serum iron level of 4.4 ± 1.2 µmol/L, a mean transferrin level of 3.06 ± 1.16 g/L, and an average total capacity of transferrin of 76.2 ± 29.1 µmol/L. The results of hemoglobin electrophoresis and the pathologies are summarized in Table 2. For the normal electrophoresis group, only hemoglobin A0 and hemoglobin A2 were detected, other fractions were not detectable.

**Table 1:** Demographic and hematological characteristics of the included patients

Parameters	Patients with normal hemoglobin electrophoresis (*n* = 13)	Patients with hemoglobin abnormalities (*n* = 17)	*p*-value
Age – median, (Q1, Q3)	37 (12–56)	27 (13–64)	0,966
Sex – N F/M	6/7 F/M	9/8 F/M	0,712
Hemoleucogram			
Red blood cells – median, (Q1, Q3)	4,39 (3,74–4,5)	3,97 (3,44–4,72)	0,7064
Hematocrite – median, (Q1, Q3)	33,4 (29,6–37,1)	32,6 (25,6–35,9)	0,346
Hemoglobin – median, (Q1, Q3)	11,1 (9,1–12,1)	10,3 (8,7–12,6)	0,8016
Mean corpuscular volume (MCV) – median, (Q1, Q3)	78,7 (73,1–89,6)	80,4 (70,05–86,9)	0,7856
Mean corpuscular hemoglobin concentration (MCHC) – median, (Q1, Q3)	32,7 (31,3–34,6)	33,9 (31,7–34,45)	0,4892

The table 1 presents the population’s statistical characteristics with the median of the values, the first and third quartile, and the p-value of the Wilcoxon–Mann–Whitney test

**Table 2:** Hemoglobin electrophoresis results

Results of hemoglobin electrophoresis	HbA	HbA2	HbAN1	HbC	HbF	HbS
Minor β- thalassemia	96,5	3,5				
Minor β- thalassemia	95,7	4,3				
Minor β- thalassemia	95,5	4,5				
β- Thalassemia intermediar	71	7,8			21,2	
Acquired elevation of HbF (anemic stress)	93,4	2,4			4,2	
Acquired elevation of HbF (anemic stress)	95,2	2,6			2,2	
Acquired elevation of HbF (diabetes)	96,2	2,7			1,1	
Composite S/C sickle cell disease	0,7	4,1		41,5	5,2	48,5
Composite S/C sickle cell disease	1	2,7		39,3	13,6	43,4
Homozygous sickle cell disease (Transfusion context)	48,2	3,4			3	45,4
Homozygous sickle cell disease (Transfusion context)	47,3	2,8			4	45,9
Homozygous sickle cell disease (Transfusion context)	0	3,1			14	82,9
HbE variant (heterozygous)	72,1	2,9	25			
HbC variant (heterozygous)	61,2	3,8		35		
Delta chain variant (heterozygous)	97,5	1,6	0,9			
Delta chain variant (heterozygous)	97,7	2,3				
Iron deficiency (5 subjects)	97,6 (97,5;98,1)	2,4 (1,9;2,5)				
Normal (9 subjects)	97,4 (97;97,6)	2,6 (2,4;2,7)				

The table 2 presents the results in hemoglobin electrophoresis in the first column and the respective percentage of hemoglobin A (HbA), hemoglobin A2 (HbA2), hemoglobin AN1 (HbAN1), hemoglobin C (HbC), hemoglobin F (HbF), and hemoglobin S (HbS). For the iron deficiency and normal patients, data are expressed as median, first and third quartile

### Comparison of the two staining methods without spiking of newborn blood

**Fig. 2:**
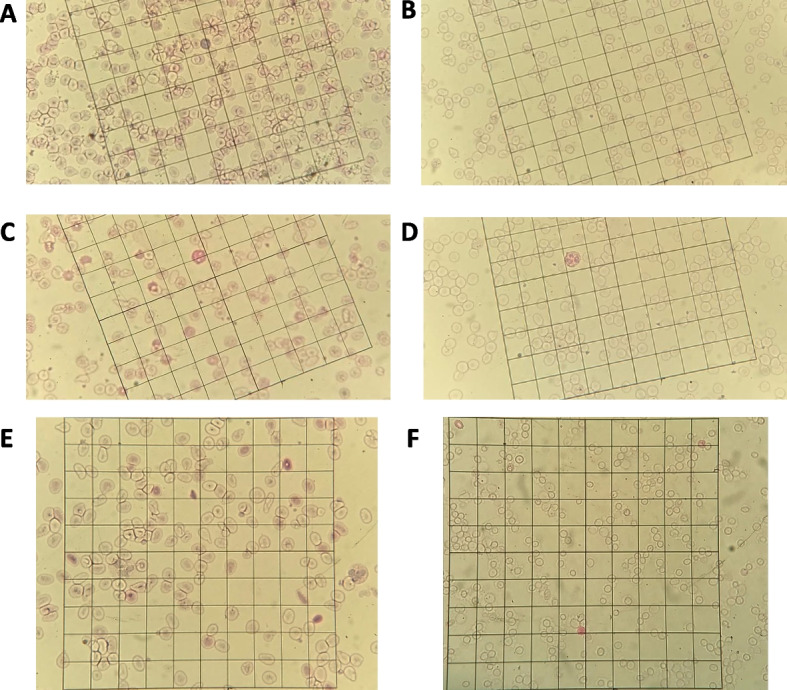
Contribution of the proposed new staining method. **A** Smear of a patient with thalassemia patient using Shepard staining. **B** Smear of the same patient using the proposed staining method. **C** Smear with a neutrophil cell that could be confused with a fetal RBC. **D** Appearance of a neutrophil cell with the new staining method. **E** Smear of a patient with sickle cell disease using Shepard staining. **F** Smear of a patient with sickle cell disease using the new staining method with the same scale as image F

From the beginning of the smear reading, we observed a high percentage of difficult slides, as expected in the chosen population. Because the slides were analyzed for one patient at a time, we concluded that, from a qualitative point of view, the slides with the new staining method were clearer; the red background was diminished, and the number of positive cells was decreased (Fig. 2A, B). Moreover, the white blood cells (neutrophil cells) were more easily recognizable with the new staining method (Fig. 2C, D). For the pathological blood slides, an improvement with the new staining procedure was observed. In addition, a decrease in the size of eluted RBCs was observed; meanwhile, the fetal RBCs, which are not eluable, retained their regular size (Fig. 2E, F). This property can be interpreted as an advantage, making the reading easier because of the increase in the size difference between adult and fetal RBCs.

**Fig. 3:**
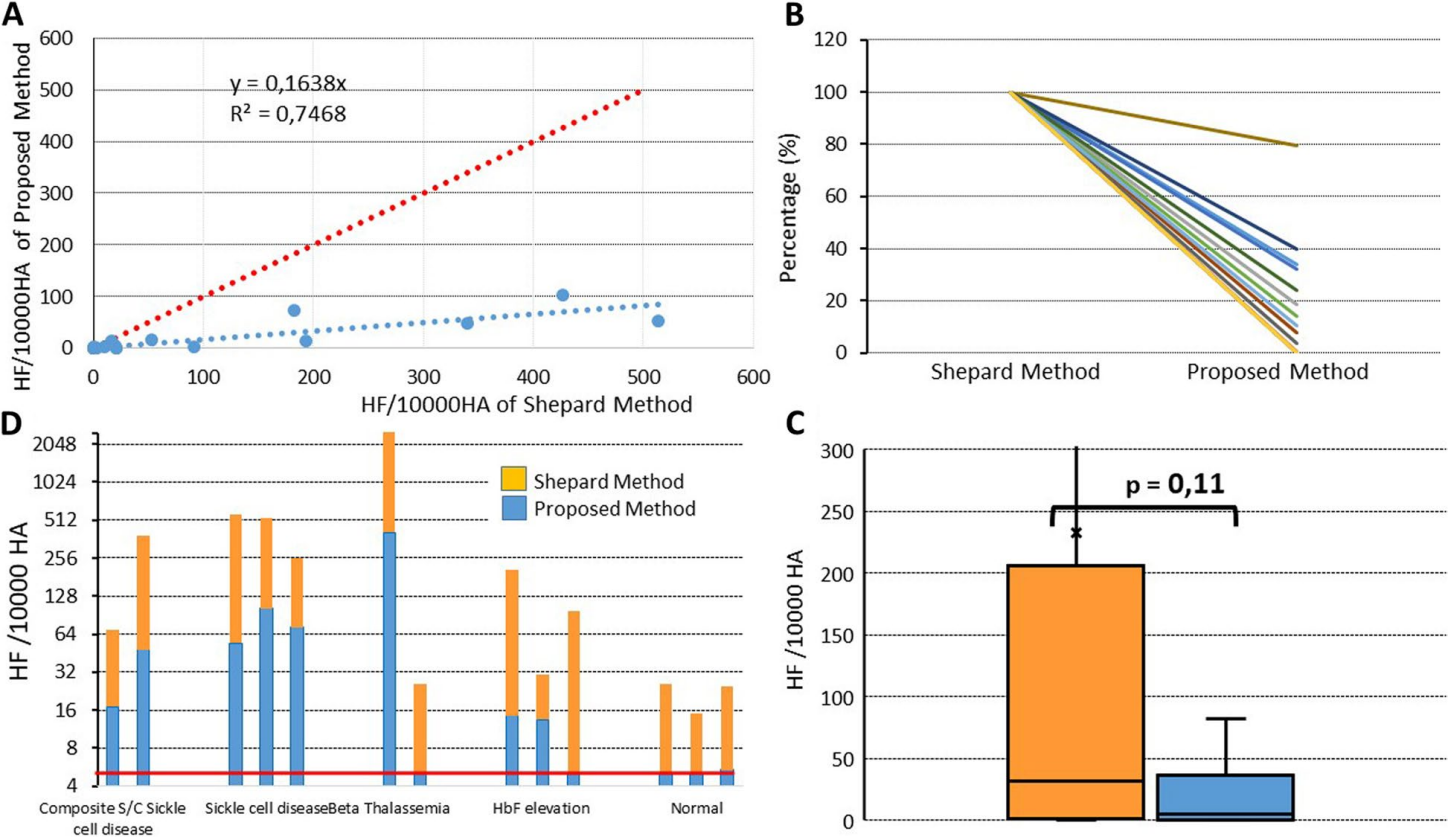
Diagnosis performance of the two staining methods. **A** Results of 30 patients (expected negative), dots having as coordinates the two results of Kleihauer test (with Shepard method and the new method); the blue line represents the trend line of the patients results, and the red line the first diagonal of the graphic. **B** Diminution of values in proposed method of Kleihauer test represented in percentage reported at the results in Sheppard method (100%) for the patients with abnormal hemoglobin electrophoresis. **C** Representation of false positive fetal RBCs/10,000 adult RBCs on a logarithmic scale; orange represents the value obtained using Shepard staining, and blue represents the value obtained using our proposed staining method. The red line is the threshold used in our laboratory. **D** The average of the differences between first and second count for the two methods, with the p result of the Wilcoxon test between the groups

The Fig. 3A represents 30 blue dots having as coordinates the two results of Kleihauer test (with Shepard method and the new method) of 30 patients (expected negative), and the blue trend line. This graph permitted the visualization of high variability of the false positive values, and it can be saw that all the values were over 6 times lower in the new method than in Shepard method. The Fig. 3B represents the selected results of the 16 samples with anomaly of hemoglobin electrophoresis and showed that the result with Shepard method decreases when the new method was used: the proposed method were always lowering the false positive results, mostly when abnormal hemoglobin was present.

The false positive cases were represented in the Fig. 3D. It was observed that when the Shepard staining was used, 10 samples (62,5%) were positive in the group of abnormal electrophoresis (sickle cell disease, thalassemia or HbF elevation) and 3 (21%) patients in the normal electrophoresis group. When the new staining was used, only 8 (50%) samples with abnormal electrophoresis were positive, and only 1 (7%) patients had slightly ambiguous cells that looked similar to newborn cells. It seems that the new staining protocol have a higher tolerance for the presence of HbS and HbF and also none of tests presents interference for HbC and HbE.

The risk of getting a false positive KB test in samples harboring abnormal hemoglobin electrophoresis was assessed by a Spearman correlation between the percentage of decreased HbA and the number of fetal RBCs counted in each method. The coefficient of correlation (r) was 0.828 (*p* < 0.001, CI 0.67—0.92) for the Shepard method and 0.615 (*p* = 0.0003, CI 0.33—0.80) for the new method, suggesting that a lower risk of a false positive KB in case of hemoglobin electrophoresis abnormality could be obtained with the new method. Thus, because the Shepard method was less performant, could make it more suitable test for hemoglobin pathologies detection.

#### Interoperator variability

The facility of lecture was represented in the Fig. 3C where the boxplots represents the difference between the first count and the second. It was observed a diminution of results variation between lectures for the proposed stain proving that this new method produces stains easier to read.

#### Accuracy of the two methods with spiking newborn blood

**Fig. 4:**
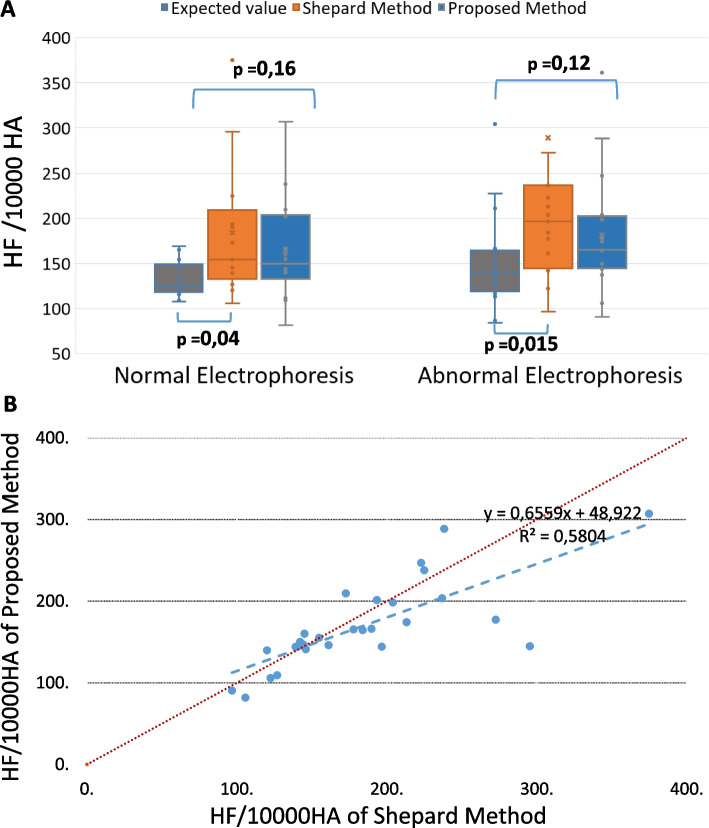
Performance of the two staining methods, 30 samples spiked with newborn blood. **A** Medians of fetal RBC counts for the two staining methods and comparison with the expected values. The graph is limited below and above at 50 and 400 fetal RBCs, respectively, per 10,000 adult RBCs. **B** Results of 30 spiked samples: dot plots having as coordinates the results of the two methods; the blue line represents the trend line of the patients results, and the red line the first diagonal of the graphic

The 30 samples spiked with newborn blood (according to the procedure described above) allowed to evaluate the accuracy of the two methods in the two groups (normal or abnormal hemoglobin electrophoresis). The Fig. 4A showed the comparison between the expected value and the results of the two methods. It was showed that in the abnormal hemoglobin electrophoresis group, the results at Kleihauer test with the proposed method were closer to the expected values than the results obtained with the Sheppard method. The Fig. 4B showed dot plots having as coordinates the results of the two methods: it can be observed an overall diminution of 11% for the values obtained with the proposed method.

#### Specificity and sensitivity of the two methods

**Fig. 5:**
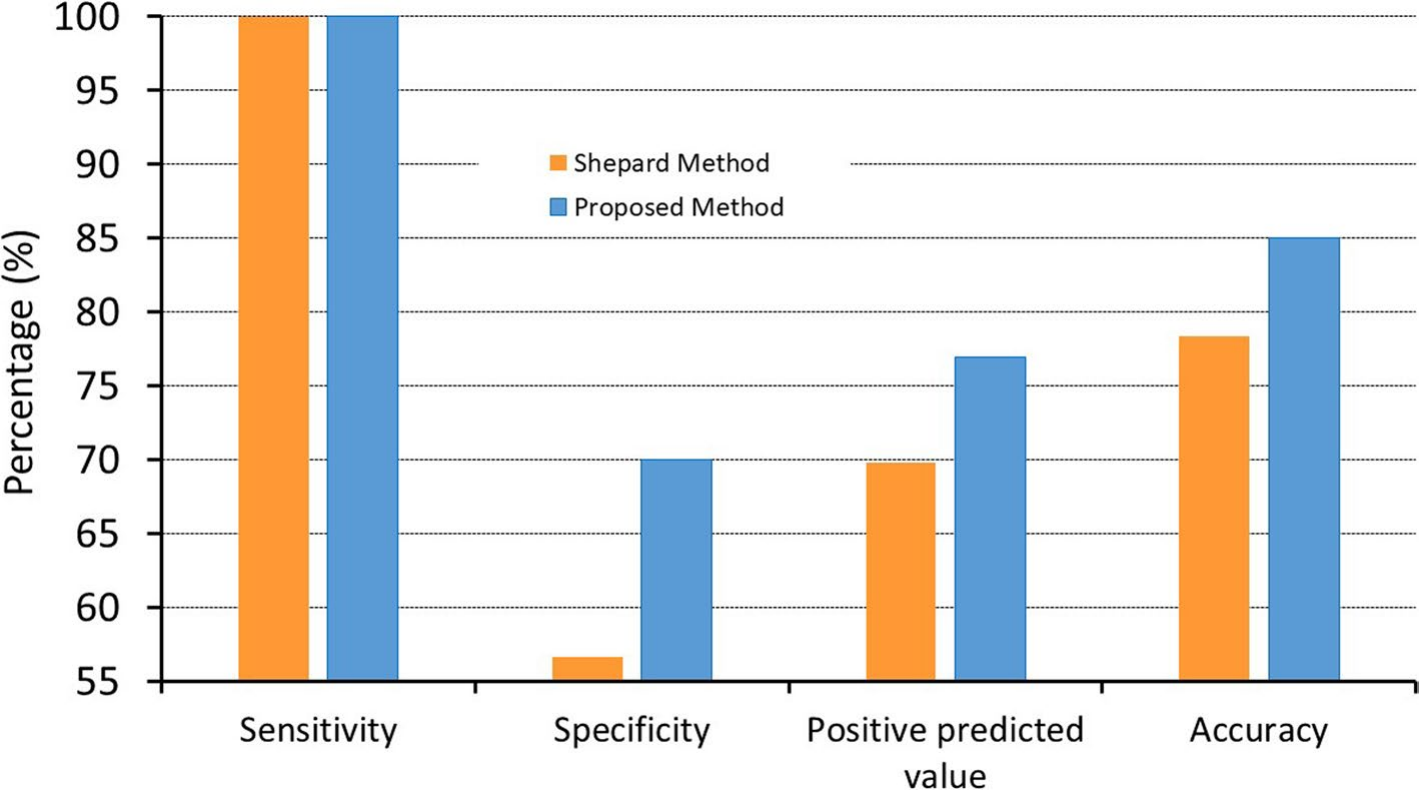
Global performance of the two staining methods. The figure depicts the performance of the two staining methods for 30 smears made with patient blood and 30 smears made with mixture of patient and newborn blood; data presented in orange refers to Shepard staining; data in blue refers to the new staining method

In total, we found that 13/30 samples (43.3%) were positive with the Shepard staining and 9/30 samples (30%) remained positive with the proposed staining (*p* = 0.29). These cases were false-positive results, because no fetal RBC were present in these 30 samples. Thus, the specificity was of 56.6% for the Shepard staining and of 70% for the new staining method (Fig. 5).

In order to calculate the sensitivity of the tests, we used the positive patient samples spiked with newborn blood (according to the procedure described above). This comparison allowed to observe veritable newborn RBC and ambiguous RBC in the same area. All the mixed samples were positive. Therefore, the sensitivity of both methods was 100% (Fig. 5), showing a very good screening performance in fetal RBC detection.

Consequently, the positive predictive value of the new stain was slightly increased reported to the positive predictive value of the Shepard stain.

We calculated the corresponding accuracy (overall probability that a patient is correctly classified) for the two stains. The performance of the new stain was overall better than Shepard stain (Fig. 5). Meanwhile, the sensitivity results could be influenced by the relatively high values of the positive cases against the positivity threshold.

### Specific cases

In two cases, the counting of slides with the patient/newborn blood mixture was impossible. In the first case (patient with minor beta thalassemia) the cells were clustered and impossible to count. We observed an important change in the slide aspect with the new staining method, which allowed the smear count and the exclusion of a technical error.

In the second case, the slides stained with Shepard method were uncountable because all the cells were broken; instead, with the new staining method, the smear was clear. To avoid a cold agglutinin disease hypothesis, we performed a direct Coombs test, which was negative. Interestingly, we found that the patient was taking disulone, an antibiotic used to treat dermatitis. The excipients used in this drug, such as iron oxalate, must have been in our opinion the source of interaction with the hydrochloric acid used in the Shepard method, and explains the absence of drug interaction in the new staining technique.

The case of subject who remain positive for both methods in the normal electrophoresis group was investigate, and we found that he had a different geographical origin (Turkey). Also, all the positive patients obtained form group of normal hemoglobin electrophoresis have submitted stress factors known to have impact on increase F hemoglobin like (diabetes, surgery, anemia). The results on hemoglobin electrophoresis represented an average of all cells contents, perhaps we could interpret these results as a temporary increase of HbF production enough to have in circulation RBC with HbF, but not enough to rise the HbF in hemoglobin electrophoresis.

Also, we could find all these stress factors in pregnancy, so this theory could explain the occurrence of ambiguous Kleihauer results. These cases highlight that we did not completely understand the hemoglobin elution process and suggested that multiple types of staining techniques could offer a solution.

## Discussion

In this study, we developed a new staining technique for the KB test that facilitates the counting of smears in difficult cases. We compared this new method with the Shepard method and demonstrated its efficacy in a population with a high number of false-positive (ambiguous) cells. In addition, the cases with (false)-positive results correlated with hemoglobin abnormalities: sickle cell disease, beta thalassemia, and HbF elevation, faking the smear reading.

Recently, the value of the KB in routine activity has been controversial [15]. A recent cohort study suggested that the outcome of a patient in an emergency does not depend on the KB results [15]; conversely, in the book [16] the ultrasonography has a low sensitivity for placental abruption and in all cases of a majour trauma KB test should be performed regardless of RH status. Also other studies have highlighted an interest in this method because it is simpler and faster than the flow cytometry technique [17–19]. Moreover, the number of weekly requests for these KB tests remains quite high in our lab. Considering these points, we propose that an improved technique is needed for this test, especially for samples that are difficult to read.

Some studies suggest that the clinical interest of a KB test in an emergency is slight [20]. Currently, clinicians have techniques such as echography, which provides a fast and precise result regarding the clinical state of the pregnancy. A fetal hemorrhage can be observed as a decrease in the blood capital of the fetus, demonstrated by a decrease in the flow characteristics of the large fetal arteries. However, the KB technique offers the advantage of quantification of the hemorrhage, allowing the adjustment of the dose of anti-D medication and the prevention of mother immunization. From another perspective, the KB technique can report the effect of a hemorrhage as an average by measuring the blood passage in a larger time interval. In addition, if a patient receives anti-D prophylaxis, we can evaluate its efficacy according to the disappearance of fetal cells. In addition, some clinicians preferred to use KB test as a confirmation, parallel to clinical and imaging tests.

In the immunohematology laboratory, we receive numerous demands for KB tests; therefore, we believe that the test is useful in clinical practice. Even the difficult KB smears are rare (several smears by month), the necessity for a new type of staining is demonstrated.

The first advantage of the new staining method is the easier counting for difficult slides. This is because of the new type of elution technique, in which the hemoglobin is dissolved and the slide is washed in a more efficient way using osmotic forces. With this elution, the RBCs that are eluted become smaller, and the cells that are not eluted become larger, making it easier to spot fetal cells. The second benefit of the new elution technique is the procedure of immersing the slide in acid for only 10 s, with the rest of the reaction occurring on the smear. The reactant is significantly less contaminated with adult hemoglobin, and the time of the elution on the smear is increased, so the eluted hemoglobin can be observed running down the smear. In addition, the fact that the eluent is less contaminated ensures lower reactant consumption and increased stability of the reactant (data not shown). Because of this property, the change in the elution reactant is not necessary as often as it is for the current technique (two weeks in our laboratory). The third benefit is the elution temperature at 37 °C, like in first technique in 1957, allowing the inactivation of cold agglutinin and other IgM, which can affect the slide assessment.

Another advantage of the new staining method is the availability of reagents, which can be easily prepared at a low cost. The blue staining reveals the nucleated cells and prevents confusion between fetal erythrocytes, reticulocytes, and small lymphocyte, which can occur with inexperienced technicians.

The first limitation of the new staining method is the longer time of the technique. Because the Shepard staining combines the elution step with the blue staining step and does not include temperature regulations, it saves time. Depending on the laboratory organization, the KB tests are typically conducted in series. In this case, the 15–20 min added in the new technique for a bank of slides is likely negligible considering the benefit that it offers. Moreover, the requirement for the elution to be performed at 37 °C may seem to require special materials, but we decided that the elution reactant could be inserted an hour before slide preparation in an incubator already present in the laboratory for microbiology or immunology purposes.

Another possible limitation of our protocol is the possible hemolysis effects and ability to disrupt cellular adhesion for the acetic acid [20, 21]. The cellular membrane already breaks during the fixation process with alcohol because the extra membrane proteins are denatured and the double lipid barrier is dissolved. The addition of acetic acid results in a second denaturation of these proteins, and we suspected that it could produce a loss of blood cells from the smear. This phenomenon was studied by changing the elution time, and we observed that a longer elution time could remove all the cells already fixed on the slide. By contrast, with a shorter elution time, the cells were not affected by acetic acid use. Therefore, from a theoretical point of view, we increased the time of cell fixation prior to elution to prevent the loss of cells during slide preparation. After 120 slides counted with the two staining methods, we can conclude that no significant cellular loss was caused by acetic acid use, and the benefit of the improved elution outweighed this possible risk.

In cases of positive KB slides, the slide must be double-counted. In cases of difficult slides, we often observe a significant difference between the two readings. Therefore, the coefficient of variation between two users can be considered a marker of the ease of slide reading. In our study, the coefficient of variation between operators was smaller in slides prepared using the new staining method than in slides prepared using the Shepard method.

The new staining method also has the benefit of increasing the specificity of data, as demonstrated in our population with a high risk of false-positives because of the presence of hemoglobin anomalies. Although the specificity was increased, we note that it is not possible to reach 100% because some hemoglobin pathologies can increase the percentage of HbF in adult patients (such as beta thalassemia, sickle cell disease, and persistence of fetal hemoglobin…). The KB test does not interfere with the presence of hemoglobin C, or E or AN1, and we cannot exclude a certain contribution of hemoglobin S to difficult smears cases.

In the newborn blood, all the RBC are filled with HbF, and it is uniformly and intensely colored on the slide. In the case of thalassemia on a KB smear, we observed that the cells staining was not uniform; the red cell took a form similar to a snowflake, suggesting that the cell charge in abnormal hemoglobin is variable. In sickle cell disease, we observed the same characteristic, but with the red pigment dissipated throughout numerous cells. In normal subjects also, HbF is present in a subsets of cells in a heterocellular manner [22], even if the hemoglobin electrophoresis is not able to show an increase in HbF. This heterogeneity is accentuated in slides with abnormal hemoglobin, explaining the difficulty of fetal RBC counting. The existence of F cells in adult blood can explain the false positivity of some of positive KB tests in non-pregnant adults [22].

An important characteristic of the KB test for both staining methods is the 100% sensitivity, which means that a positive patient is never reported as a false negative. For the clinician, this property ensures that there is no risk of underestimation of fetal–maternal bleeding.

In our study, the assessment of accuracy indicated that both techniques overestimated the number of fetal RBCs, but the new technique overestimated it less. In addition, the specificity was lowered by the new staining protocol in the chosen population. Likewise, the small number of patients is a limitation of our study. Therefore, large studies with more patients are required to study the hemoglobin elution further and determine why difficult slides appear.

Considering the advantages and limitations of the new staining method, we propose that the new technique should be used especially in difficult cases, as it is faster and cheaper than flow cytometry [23]. For routine cases, the Shepard method is an excellent tool; it is fast with sufficient performance for current clinical demands.

In conclusion, we believe that the automation of slide preparation and reading will be more suitable with the new technique because of the reduced red background on the smear, the increased brightness of white cells, and the size difference between the fetal and adult cells. This automated counting can be achieved by using a digital microscope to scan the slide, sorting the cells, and identifying them; using technology already available in hematology laboratories; or by simple evaluation of the red signal on the slide because of the increased contrast offered by our proposed staining.

## Conclusion

The new staining method was demonstrated to be a suitable technique for difficult KB slides, reducing the number of false-positive results and making the slides easier to read. Because of the time-consuming staining, we propose that the new technique should be used as a complement to the laboratory’s current technique and needs to be studied further in a larger population.

### Supplementary Information

**Additional file 1.**

## Data Availability

The dataset used in the present study is available from the corresponding author upon reasonable request.
