# Correction: Development of a new staining protocol for the Kleihauer–Betke test to facilitate the reading of difficult cases

**DOI:** 10.1186/s12884-024-06333-1

**Published:** 2024-02-13

**Authors:** Adrian Serban, Yannick Tholance, Carmen Aanei, Lydia Campos, Cristina Iobagiu

**Affiliations:** 1 Laboratory of Biochemistry, University Hospital of Saint-Etienne, Saint-Priest-en-Jarez, 42275 France; 2 Institute NeuroMyoG?ne, INSERM U1217/CNRS UMR 5310, Universit? Claude Bernard Lyon 1, Lyon, France; 3 Laboratory of Hematology, University Hospital of Saint-Etienne, Saint-Priest-en-Jarez, 42275 France; 4 INSERM U1059-SAINBIOSE, Universit? de Lyon, Saint-Etienne, France; 5 Immunohematology Laboratory, Etablissement Fran?ais de Sang, Saint-Etienne, France

**Correction: BMC Pregnancy Childbirth 24, 89 (2024)**

10.1186/s12884-024-06258-9

Following publication of the original article [1], the authors identified an error in the author names. The given names and family names were erroneously transposed.

The incorrect author names are: **Serban Adrian, Tholance Yannick, Aanei Carmen, Campos Lydia, Iobagiu Cristina**.

The correct author names are: **Adrian Serban, Yannick Tholance, Carmen Aanei, Lydia Campos, Cristina Iobagiu**.

Also, the statement “The financial support was ensure by the Foundation ‘Les ami de Remi’” in Funding section was removed due to the funding was not concluded.

The author group has been updated above and the original article [1] has been corrected.
